# Development and application of therapeutic antibodies against COVID-19

**DOI:** 10.7150/ijbs.59149

**Published:** 2021-04-10

**Authors:** Lin Ning, Hamza B. Abagna, Qianhu Jiang, Siqi Liu, Jian Huang

**Affiliations:** 1 School of Healthcare Technology, Chengdu Neusoft University, Sichuan, China; 2 School of Life Science and Technology, University of Electronic Science and Technology of China, Sichuan, China; 3 Center for Informational Biology, University of Electronic Science and Technology of China, Sichuan, China

**Keywords:** therapeutic antibody, COVID-19, SARS-CoV-2, convalescent plasma, antibody cocktail, monoclonal antibody

## Abstract

The pandemic of Coronavirus disease 2019 (COVID-19) caused by the severe acute respiratory syndrome 2 coronavirus (SARS-CoV-2) continues to be a global health crisis. Fundamental studies at genome, transcriptome, proteome, and interactome levels have revealed many viral and host targets for therapeutic interventions. Hundreds of antibodies for treating COVID-19 have been developed at preclinical and clinical stages in the format of polyclonal antibodies, monoclonal antibodies, and cocktail antibodies. Four products, *i.e.*, convalescent plasma, bamlanivimab, REGN-Cov2, and the cocktail of bamlanivimab and etesevimab have been authorized by the U.S. Food and Drug Administration (FDA) for emergency use. Hundreds of relevant clinical trials are ongoing worldwide. Therapeutic antibody therapies have been a very active and crucial part of COVID-19 treatment. In this review, we focus on the progress of therapeutic COVID-19 antibody development and application, discuss corresponding problems and challenges, suggesting new strategies and solutions.

## Introduction

The Coronavirus disease 2019 (COVID-19) is an emerging infectious disease caused by the severe acute respiratory syndrome 2 coronavirus (SARS-CoV-2) 1,2. The clinical outcome of SARS-CoV-2 infection ranges from asymptomatic, flu-like symptoms, and pneumonia to acute respiratory distress syndrome (ARDS), renal failure, and other deadly complications 3. On March 11, 2020, the World Health Organization (WHO) declared the COVID-19 outbreak a global pandemic 4. As of January 31, 2021, 102,083,344 confirmed cases of COVID-19, including 2,209,195 deaths, were reported to WHO 5. To defeat the ongoing COVID-19 pandemic, worldwide research efforts have been made at genome, transcriptome, proteome, and interactome levels to help understand the mechanism of the disease and develop efficient vaccines and drugs, including therapeutic antibodies.

## Potential Targets for Developing COVID-19 Therapeutic Antibodies

**Figure 1:**
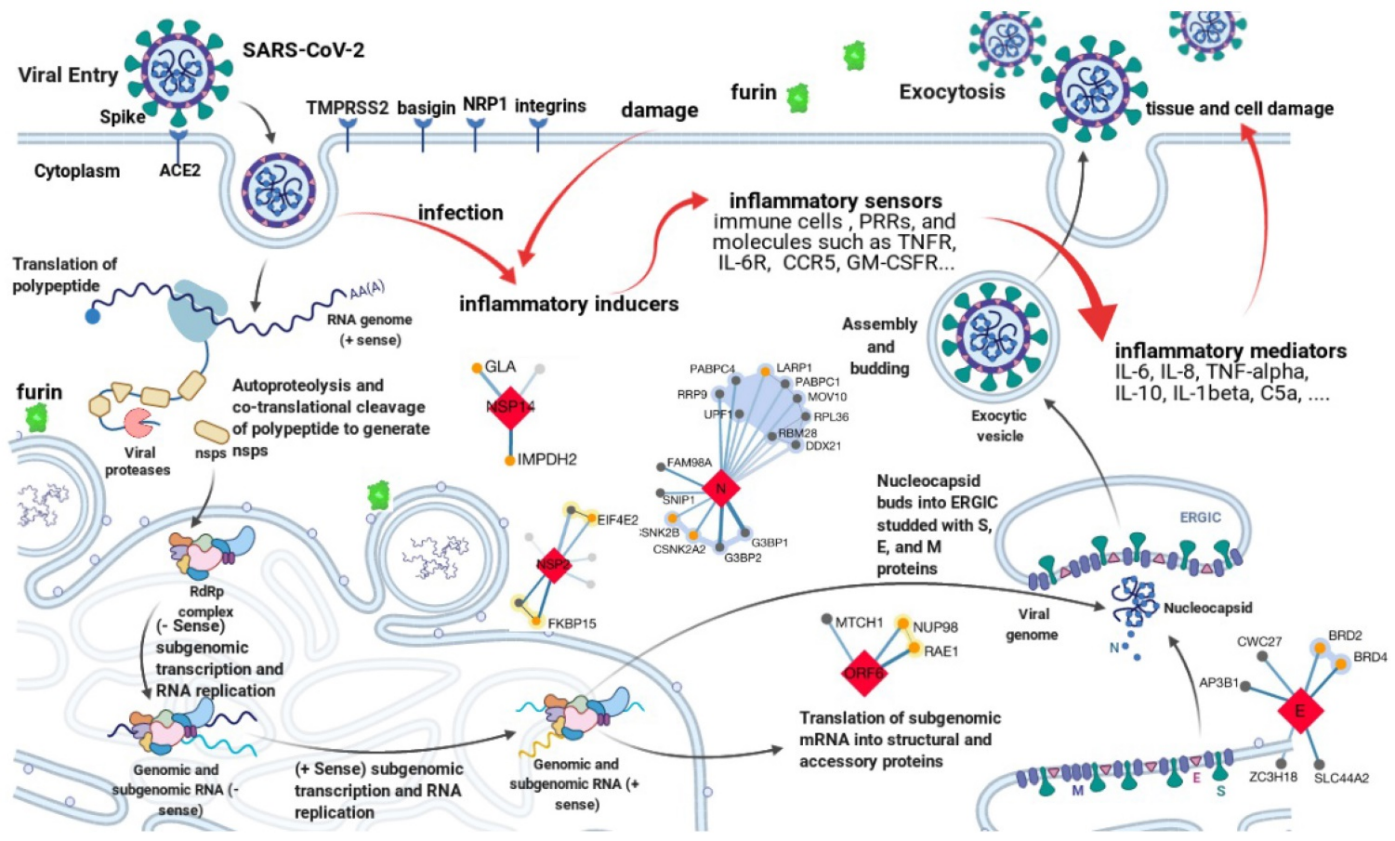
Potential targets for antibody development against COVID-19. The life cycle of SARS-CoV-2 and the complex virus-host interactions revealed by genomics, transcriptomics, proteomics, and interactomics studies present various potential targets for therapeutic interventions. All targets can be grouped into two categories. One is the anti-virus category, such as antibodies target the spike protein to block viral entry. Another is the anti-host category, such as levilimab targets IL-6R to inhibit inflammation. The figure is created with BioRender.com using its editable templates 6.

Genomics studies have shown SARS-CoV-2 is a novel member of the genus betacoronavirus. It has a positive-sense, single-stranded RNA genome about 30kb long 2. As with all other coronaviruses, genes of SARS-CoV-2 are grouped into 3 categories, *i.e.*, nonstructural, structural, and accessory. The nonstructural gene ORF1ab occupies about 2/3 of the genome at the 5′ end, encoding polypeptides pp1a and pp1ab (-1 ribosomal frameshift). The proteolytic cleavage of pp1a and pp1ab produces a series of nonstructural proteins (namely nsp1-16), which are essential for viral transcription and replication 6. The left 1/3 of the genome at the 3′ end accommodates structural and accessory genes. There are 4 structural genes, namely S, E, M, and N, encoding Spike, Envelope, Membrane, and Nucleocapsid protein respectively. The N protein is responsible for viral genome packing; the M protein plays a central role in viral morphogenesis, assembly, and egress; the E protein is critical for viral envelope curvature, maturation, and budding 6. The S protein has two subunits, S1 and S2, determining viral entry. S1 contains the receptor-binding domain (RBD) and directly contacts with the host receptor, angiotensin-converting enzyme 2 (ACE2), whereas S2 mediates the following membrane fusion and enables the virus to enter the host cytoplasm 7-11. The accessory genes include ORF3a, ORF6, ORF7a, ORF8, *etc* 12. Although not essential for viral replication or structure, their products can modulate host innate or adaptive immune response and play an important role in viral pathogenicity. As shown in Figure 1, all proteins encoded by the SARS-CoV-2 genome are potential anti-virus targets for developing therapeutic antibodies against COVID-19. Among them, the S protein is the major target for neutralizing antibody development due to the mechanism of blocking viral entry.

Transcriptomics, proteomics, and interactomics studies have further revealed many other potential drug targets for COVID-19 therapeutics development 13-19. Hundreds of interactions between human and SARS-CoV-2 proteins are identified 16-19. For example, besides ACE2, human transmembrane serine protease 2 (TMPRSS2) 20, furin 21, basigin 22, neuropilin-1 23, and integrins 24 are also involved in SARS-CoV-2 entry. Immune response to SARS-CoV-2 is an important part of the complex virus-host interactions 3. It is, however, a double-edged sword. The innate and then the following adaptive immune response after SARS-CoV-2 entry, on one hand, will help clear the virus and block further infection. On the other hand, high levels of inflammatory cytokines and chemokines such as tumor necrosis factor-alpha (TNF-α), interleukin-6 (IL-6), interleukin-8 (IL-8), and interleukin-10 (IL-10) can amplify tissue damage and even cause cytokine storm, leading to respiratory/organ failure 25-27. As shown in Figure 1, all host proteins involved in virus-host interactions or significantly perturbed by SARS-CoV-2, especially factors relevant to the inflammatory response pathway are also potential targets for therapeutic interventions against COVID-19. The main mechanism of these host targets is anti-inflammation.

A lot of studies on chemical drug design or repurposing for the treatment of COVID-19 have been done 28-33. However, only dexamethasone resulted in lower 28-day mortality in a controlled, open-label trial 34. All other drugs such as remdesivir, hydroxychloroquine, lopinavir, and ritonavir had little or no effect on hospitalized COVID-19 patients, with regards to overall mortality, hospital stay duration, and initiation of ventilation 35. Current treatment for COVID-19, therefore, is still mainly based on supportive and symptomatic care. Fortunately, all kinds of therapeutic antibodies have also been developed and applied in hundreds of clinical trials, showing promising results 36-45. This review focuses on the development and application of therapeutic antibody therapies for COVID-19 in polyclonal, monoclonal, and cocktail format, addressing relevant progress, problems, challenges, possible strategies, and solutions.

## Therapeutic Polyclonal Antibodies

Therapeutic polyclonal antibodies have been applied to treat infectious diseases since the 1890s. In 1901, Emil Behring received the first Nobel Prize in Physiology or Medicine for his work on serum therapy, especially its application against diphtheria. Later on, convalescent sera or plasma therapies have been applied to various infectious diseases. Due to the increasing number of SARS-CoV-2 infections and the lack of effective therapies, convalescent plasma therapy has become very popular currently.

According to data from ClinicalTrials.gov, there are at least 176 registered clinical trials using convalescent plasma for treating COVID-19 patients. Although most of these clinical trials are still ongoing, there are 26 completed studies. Some early results show convalescent plasma therapy is not only safe but also may help eliminate the virus and improve clinical symptoms 41,43-45. On August 23, 2020, the U.S. Food and Drug Administration (FDA) authorized the emergency use of convalescent plasma for the treatment of hospitalized patients with COVID-19. However, some newly released results from randomized and controlled trials seem controversial about the efficacy and benefits of convalescent plasma.

A meta-analysis on 20 studies (including one randomized controlled trial) concluded that the benefits of convalescent plasma for people admitted to hospital with COVID-19 were uncertain 46. An open-label, multicenter, randomized clinical trial was performed in 7 medical centers in China (Chinese Clinical Trial Registry: ChiCTR2000029757). Its results showed that convalescent plasma therapy added to standard treatment did not result in a statistically significant clinical improvement within 28 days in patients with severe or life-threatening COVID-19 47. The results of an open-label, multicenter, randomized clinical trial (PLACID Trial) in India showed no difference in 28-day mortality or progression to severe disease between patients with moderate COVID-19 treated with convalescent plasma and control group 48. The median time from symptom onset to enrollment in this trial was 8 days 48. A double-blind, placebo-controlled, multicenter trial was conducted at 12 sites in Argentina (ClinicalTrials.gov number, NCT04383535). A total of 228 patients got convalescent plasma and 105 patients received a placebo (normal saline solution) in addition to standard treatment. No significant differences were observed in clinical status or overall mortality between the plasma group and the placebo group over 30 days 49. Interestingly, the median time from the onset of symptoms to enrollment in the trial was also 8 days 49.

More and more positive results however have also been reported recently. In a retrospective, propensity score-matched, case-control study, the effectiveness of convalescent plasma therapy in 39 patients with severe or life-threatening COVID-19 at the Mount Sinai Hospital in New York City was assessed. The median time between admission and transfusion was 4 days. Both oxygen requirements and survival were improved in plasma recipients 50. A prospective, propensity score-matched study compared the efficacy of COVID-19 convalescent plasma with the standard of care. The data showed a significant decrease in mortality within 28 days, specifically in patients transfused within 3 days of admission with plasma with an anti-RBD titer of ≥1:1350 51. This study suggests that COVID-19 convalescent plasma treatment with high anti-RBD IgG titer is efficacious in early-disease patients 51. Further, the results from a 60-day follow-up implied an optimal window of 44 hours after hospitalization for transfusing COVID-19 patients with high-titer convalescent plasma 52. Appropriate COVID-19 convalescent plasma therapy can not only reduce mortality but also the progression of COVID-19. In a very recent report on a randomized, double-blind trial, 250 milliliters of convalescent plasma with an anti-SARS-CoV-2 S protein IgG titer greater than 1:1000 was compared with saline placebo in older patients 53. All patients in the trial received convalescent plasma or placebo less than 3 days after symptom onset. About 16% of patients progressed to severe COVID-19 in the convalescent plasma group, whereas the ratio of the placebo group was as worse as 31%. Also, a dose-dependent effect related to the antibody titer after the infusion was observed. The authors concluded that early infusion of high-titer convalescent plasma to mildly ill infected older patients can reduce the progression of COVID-19 53. The expanded access program of Mayo Clinic has treated more than 70,000 patients with convalescent plasma. An antibody titer-dependent effect was found again. In a retrospective study based on Mayo's data, a lower risk of death within 30 days in the high-titer group than in the low-titer group was observed among patients who had not received mechanical ventilation before plasma transfusion 54.

Taking the seemingly contradictory results from clinical trials together, it becomes clear that the use of convalescent plasma with high anti-RBD IgG levels at the early stage (preferably within 3 days from the onset of symptoms) can reduce mortality and progression of COVID-19 55. Although the FDA authorized both high titer and low titer COVID-19 convalescent plasma for emergency use, it has revised the guidance and reissued the EUA for COVID-19 convalescent plasma very recently because the efficacy of low titer plasma may be compromised or doubtful. For patients with severe or life-threatening COVID-19, the benefits of convalescent plasma therapy are still uncertain.

The major problem with convalescent plasma therapy is quality control and standardization. For example, the antibody titer of plasma is highly variable, but no measurement for neutralizing antibodies is widely available or generally accepted, though FDA has set a standard to define high titer convalescent plasma (*i.e.*, Ortho VITROS SARS-CoV-2 IgG tested with signal-to-cutoff ratio ≥12). Furthermore, the optimal dose and time point, as well as the most proper patients, still need further investigations, especially in the situation where the supply of convalescent plasma is tenuous compared to a large number of patients. Thirdly, the risk of infecting unknown blood-borne infectious diseases exists, though strict screen is performed before the collection of plasma. Besides, convalescent plasma is not easy to store and deploy compared with other drugs.

To deal with the above problems, intravenous immunoglobulin (IVIG) has also been proposed to treat COVID-19 56,57. IVIGs are sterile, purified immunoglobulins (typically more than 95% unmodified IgG) products from the plasma of approximately a thousand or more blood donors. It is available as either a liquid or lyophilized powder; the latter is very convenient to store and transfer. As our world is far from herd immunity, most plasma donors are not likely to contain antibodies against SARS-CoV-2. Therefore, current case reports and clinical trials are insufficient to support the efficacy of IVIG without specific anti-SARS-CoV-2 IgG 58,59. Nevertheless, with the ongoing pandemic and the continuing lack of effective chemical drugs, convalescent plasma therapy need to learn from IVIG with respect to standardization, quality control, and manufacture. It is expected that convalescent plasma therapy can evolve from a stopgap treatment to an IVIG drug with SARS-CoV-2-Specific immunoglobulins, which can be collected from donors recovered from COVID-19 or successfully immunized with COVID-19 vaccine.

## Therapeutic Monoclonal Antibodies

Therapeutic monoclonal antibodies have been applied to treat human diseases since 1986 when muromonab-CD3 was approved by FDA for treating acute rejection after a kidney transplant 60. It has rapidly become a major part of the pharmaceutical industry for the past 35 years. At present, its annual market reaches 150 billion US dollars. It is widely used in the fight against cancer, inflammatory and autoimmune diseases. Therapeutic monoclonal antibodies have also been used to treat infectious diseases. Palivizumab is the first monoclonal antibody approved for infectious disease. In 1998, the FDA authorized palivizumab to prevent serious lung disease caused by the respiratory syncytial virus in infants 61. Since then, more and more therapeutic monoclonal antibodies have been developed for quite a few infectious diseases, including some emerging infectious diseases 62,63. The development of therapeutic monoclonal antibodies is currently at the front line of fighting against the COVID-19 pandemic 36. According to their targets, we divide the COVID-19 therapeutic monoclonal antibodies into anti-virus and anti-host categories.

As described previously, the spike protein of SARS-CoV-2 mediates the virus entry and the effect of convalescent plasma therapy is dependent on the titer of neutralizing anti-spike antibodies. It is therefore only natural that the main target of anti-virus antibodies is the spike protein. In the last year, a lot of research groups have isolated many monoclonal antibodies against the spike protein of SARS-CoV-2, especially against its receptor-binding domain 64-88. Most of them are human antibodies, while some are from camelid 85, alpaca 87, and llama 88. The human antibodies are selected from genetically humanized mice or convalescent patients using hybridoma, phage display, and single B cell technology. Most antibodies are IgG, while other classes exist such as IgA 80. These antibodies are usually SARS-CoV-2 specific, while some can also neutralize SARS-Cov 65,68,72,80,82. A lot of *in vitro* and *in vivo* experiments have been done in the preclinical researches to study epitopes, structures, efficacy, and mechanisms of anti-spike monoclonal antibodies 38. Many promising anti-virus monoclonal antibodies have gone into clinical trials 89. As shown in Table 1, there are now 3 anti-spike monoclonal antibodies at the stage of phase 3 clinical trials 40. In an interim analysis of a phase 2 trial, the neutralizing antibody bamlanivimab appeared to accelerate the natural decline in viral load of mild or moderate COVID-19 outpatients at the dose of 2800mg 90. On November 9, 2020, the FDA issued bamlanivimab an emergency use authorization (EUA) for the treatment of recently diagnosed mild to moderate COVID-19 in patients who are older than 12 years old, weigh at least 40 kg, and are at high risk of progressing to severe disease and/or hospitalization 91. Another way to target the spike protein is to engineer human ACE2 with Fc fragment of antibody 92-95. Other SARS-CoV-2 proteins such as envelope, membrane, and nucleocapsid proteins are also essential to the virus. For different purposes such as research and diagnosis, lots of monoclonal antibodies against these targets are also being and have been developed 96.

Host factors involved in the life cycle of SARS-CoV-2 or the pathogenesis of COVID-19 are also potent targets for therapeutic monoclonal antibody development. For example, neuropilin-1 (NRP1) significantly increases SARS-CoV-2 infectivity and mAb3, a monoclonal blocking antibody against NRP1 can block this effect in cell culture 97. Basigin (BSG, CD147, EMMPRIN) is also found to involve in the entry of SARS-CoV-2 22. Recently, a humanized anti-CD147 monoclonal antibody called meplazumab has been repurposed to treat COVID-19. The preliminary experimental and clinical findings are very positive 98,99. As CD147 also plays a role in the inflammatory response, the monoclonal antibodies against this target may not only have an anti-virus effect (help block the viral entry) but also exert an anti-inflammation effect. To deal with inflammation, cytokine storms, and other complications caused by SARS-CoV-2 infection, a lot of anti-host monoclonal antibodies have been repositioned to treat COVID-19 (see Table 2). For example, tocilizumab, a monoclonal antibody targeting IL-6R, originally approved by the FDA for use in patients with rheumatologic disorders, has been used to treat COVID-19 in several trials. The preliminary data from the Chinese population show that tocilizumab improves the clinical outcome in severe and critical COVID-19 patients, implying an effective treatment to reduce mortality 42. A report from Italy concludes tocilizumab may reduce the risk of invasive mechanical ventilation or death in patients with severe COVID-19 100. Two recent reports add that tocilizumab is not effective for preventing intubation or death in moderately ill hospitalized COVID-19 patients 101, but can reduce the likelihood of progression to the composite outcome of mechanical ventilation or death in hospitalized COVID-19 patients without mechanical ventilation although do not improve survival 102.

Taking the two categories together, there are hundreds of therapeutic monoclonal antibodies at the different stages of development. The community does need a special database that can aggregate and store COVID-19 antibody data together, and most importantly, easily follow the progress of relevant projects. In March 2020, the Chinese Antibody Society launched the “COVID-19 Antibody Therapeutics Tracker” project to track the therapeutic antibodies for COVID-19 treatment in preclinical and clinical development worldwide 40. Currently, in the database, there are 217 antibody programs against 62 targets. Among them, 133 programs are targeting the spike protein. There are 79 antibody programs in clinical trials, including 25 programs that target the spike protein.

Compared with therapeutic polyclonal antibodies such as convalescent plasma which is highly variable and heterogeneous, a monoclonal antibody drug is composed of totally identical antibodies. The monoclonal antibody usually has only one drug target and recognizes only one epitope. It can be precisely engineered and optimized to serve specific treatment purposes. Therefore, it is usually safer and more effective than polyclonal antibodies. Furthermore, the quality of monoclonal antibody is easier to control, the manufacture of monoclonal antibody is scalable and independent of donors, and the effect of monoclonal antibody is highly reproducible. However, therapeutic polyclonal antibodies are more robust and resistant to SARS-Cov-2 mutations and variants, because they have multiple drug targets and bind even more epitopes. Thus, a new format that combines the advantages of both therapeutic polyclonal antibodies and therapeutic monoclonal antibodies is urgently needed.

## Therapeutic Cocktail Antibodies

Therapeutic cocktail antibodies are combinations of two or more monoclonal antibodies. As all its components are clearly identified and characterized, an antibody cocktail holds all advantages of monoclonal antibodies. Moreover, it targets more than one epitope or even binds multiple antigens like polyclonal antibodies. The synergism and complementarity of each monoclonal antibody make a cocktail a better choice for treating varying infectious diseases 103. For example, a combination of three monoclonal antibodies called Zmapp exceeds the efficacy of any other therapeutics against the Ebola virus, including the Guinean variant of Ebola 104.

Therapeutic cocktail antibodies have become the frontiers of COVID-19 treatment. Some primary data show that cocktail treatment is superior to monotherapy. Bamlanivimab has shown promising interim results in the phase 2 trial and has been approved by the FDA for emergency use. Nevertheless, the viral load reduction of the bamlanivimab monotherapy is not significantly different from the placebo group in a randomized clinical trial. However, a statistically significant reduction in SARS-CoV-2 viral load at day 11 is observed when nonhospitalized patients with mild to moderate COVID-19 are treated with the combination of bamlanivimab and etesevimab 105. Etesevimab, also known as JS016 or LY-CoV016, is a fully human monoclonal neutralizing antibody that specifically binds the RBD of SARS-CoV-2 spike protein 77. The two monoclonal antibodies both target the spike protein of the virus, but recognize two different epitopes. Very recently, an EUA has been issued for bamlanivimab and etesevimab administered together for the indication same to that of bamlanivimab.

**Figure 2:**
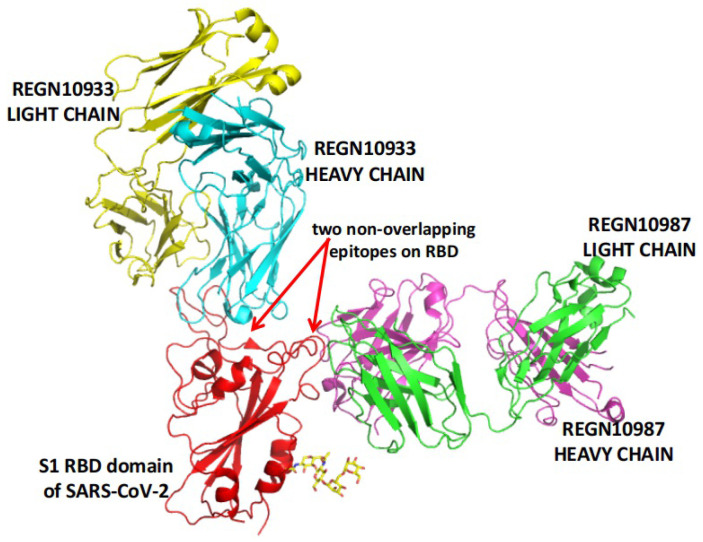
REGN-COV2 binds two non-overlapping epitopes on the spike protein of SARS-CoV-2. The image is produced with PyMOL based on the PDB structure 6XDG 73.

REGN-COV2 is a cocktail of two potent neutralizing antibodies (REGN10933/Casirivimab and REGN10987/Imdevimab). As shown in Figure 2, the two antibodies also target two distinct, non-overlapping epitopes on the spike protein of SARS-CoV-2. In animal models using rhesus macaques and golden hamsters, REGN-COV-2 can greatly reduce virus load, limit weight loss, and alleviate pneumonia, providing strong evidence for clinical trial 107. In an ongoing multicenter, double-blind, randomized, placebo-controlled, phase 1-3 trial involving nonhospitalized COVID-19 patients, the interim analysis concludes that the REGN-COV2 antibody cocktail can reduce viral load. A greater effect can be seen in patients who had a high viral load at baseline or whose immune response has not yet been initiated 108. On November 21, 2020, the FDA issued an EUA for REGN-COV2 for the treatment of mild to moderate COVID-19 in adults and pediatric patients (12 years of age or older weighing at least 40 kilograms) with positive results of direct SARS-CoV-2 viral testing and who are at high risk for progressing to severe COVID-19. This includes those who are 65 years of age or older or who have certain chronic medical conditions.

Indeed, when two epitopes are not overlapping, relevant antibodies complement rather than compete with each other 106. COV2-2196 and COV2-2130 are two potently neutralizing monoclonal antibodies recognizing two non-overlapping epitopes. They bind simultaneously to the spike protein and then synergistically neutralize wild-type SARS-CoV-2 virus 106. The two antibodies are further optimized by AstraZeneca and then made the cocktail named AZD7442 (AZD8895/Tixagevimab and AZD1061/Cilgavimab) 109, which is in phase 3 clinical trials.

At present, several other monoclonal antibody cocktails such as BRII (BRII-196 and BRII-198) and ADM03820 (a 1:1 mixture of two non-competitive bindings, human IgG1 monoclonal antibodies) are in clinical trials. As shown in Table 3, therapeutic cocktail antibodies for COVID-19 treatment are summarized. All currently known cocktail antibodies target the spike protein of SARS-CoV-2.

## Challenges and Perspectives

Antibody-dependent enhancement (ADE) is an unexpected phenomenon that happened after vaccination or antibody therapies, where the production or presence of specific antibodies may enhance rather than inhibit viral infection 110. ADE has been observed in over 40 kinds of viruses, including two well-known coronaviruses: SARS-CoV and MERS-Cov 110. A common mechanism of ADE is that viral-specific antibody promotes viral entry into host granulocytes, monocytes, macrophages, dendritic cells, and B cells through the Fc receptor (FcR) and complement receptors 110,111. ADE in SARS-CoV-2 has not yet been validated experimentally, but it may exist. Human lymphoid tissues and many immune cells usually lack ACE2 expression 112. A recent single-cell RNA sequencing study with 284 samples from 196 COVID-19 patients and controls has created a comprehensive immune landscape with 1.46 million cells. The data however reveal that SARS-CoV-2 RNAs exist in many immune cell types, including granulocytes, macrophages, plasma cells, T cells, and Natural Killer cells, indicating ADE or new routes for the virus entry other than ACE2 receptor 113. ADE has two faces. One is bad, injuring immune cells, amplifying the infection, and triggering harmful immunopathology. Another may be good, promoting antigen presentation and protective immune response. However, the bad one has become a common challenge for the development of vaccines and antibody therapies 114,115. This is especially true for vaccine development, where the immune response largely relies on the genetic background of individuals and is hard to predict. For antibody development, *in vitro* assays and *in vivo* models for ADE risk evaluation are expecting to be built. As monoclonal antibodies are easy to be engineered, quite a few strategies such as Fc engineering and antibody cocktails may bypass or inhibit ADE.

The second challenge comes from SARS-CoV-2 variants 116-118, as mutations might yield antibody resistance. Experiments show that SARS-CoV-2 variants with mutations in the RBD or N-terminal domain of the spike protein and then resistance to monoclonal antibodies or convalescent plasma can be readily selected 117. The emergence of antibody-resistant SARS-CoV-2 variants may limit the efficacy of therapeutic monoclonal antibodies. This is confirmed in another study, where novel spike mutants rapidly appear after *in vitro* passaging in the presence of individual antibodies, resulting in loss of neutralization 119. A proper antibody cocktail can be a solution to this challenge. After treatment with a noncompeting antibody cocktail (REGN-COV2), escape mutants were not generated. However, using an antibody cocktail (REGN10989+REGN10934) in which the components showed complete competition, rapid escape occurred and ablated neutralization of the cocktail. For another cocktail (REGN10989+REGN10987) in which the components exhibited only partial competition, such rapid escape was not observed 119. Another cocktail strategy is to add a conservative and cross-neutralizing monoclonal antibody in the combination. S309 is a monoclonal antibody selected from memory B cells of one SARS patient, which can also neutralize SARS-CoV-2. Antibody cocktails using S309 in combination with other antibodies can enhance SARS-CoV-2 neutralization and limit the emergence of neutralization-escape mutants 65,120. Currently, all antibody cocktails for treating COVID-19 are combinations of monoclonal antibodies targeting different epitopes on the SARS-CoV-2 spike protein. We expect new cocktails could extend to various targets rather than the spike protein only. For example, can we combine anti-virus and anti-host monoclonal antibodies together? Or can we utilize ADE to let antibodies also work in the cell? Thus, new cocktails can target the spike protein as well as envelope, membrane, nucleocapsid, and other proteins.

The third challenge is how to survive drowning in the sea of COVID-19 data and papers 121. A simple search with the keyword “COVID-19” against the MEDLINE database returns more than 100,000 papers at the time of writing this review. The situation is true for antibody development against COVID-19 too. We conducted a bibliometric study to gain a better understanding of the trend for antibody development against COVID-19 using the Web of Science database. A total of 4,435 publications related to therapeutics antibodies against COVID-19 were found. In fact, no one has enough time to read through all existing papers or even keep up with all new data and papers. To read and write reviews is a traditional, yet effective and valuable solution to this challenge. Furthermore, bioinformatics and artificial intelligence can help 122. For the special information on therapeutic antibodies for COVID-19 treatment, the COVID-19 antibody therapeutics tracker is a very good attempt and example to this challenge 40. However, it lacks antibody sequence and structure, which are still dispersed in papers and other databases, *e.g.* the PDB database. As the information mentioned above is essential for antibody analysis and evaluation, a more comprehensive database for therapeutic antibodies against COVID-19 is needed. Facing hundreds and even thousands of antibody candidates, better and more bioinformatics tools for evaluating the antibody developability are also needed to accelerate the speed of antibody development 123,124.

## Conclusion and Future Aspects

The available therapeutic antibodies for COVID-19 treatment can be divided into three categories: polyclonal, monoclonal, and cocktail antibodies. Four products have received EUA from the FDA, *i.e.* convalescent plasma, bamlanivimab, REGN-Cov2, and the combination of bamlanivimab and etesevimab. Currently, hundreds of therapeutic antibodies against COVID-19 are at the preclinical stage or in clinical trials, making therapeutic antibodies an important complement to interventions such as chemical drugs and vaccines.

The development of antibody therapeutics for COVID-19 is challenged by the risk of antibody-dependent enhancement, SARS-CoV-2 variants, and information overload. Future studies should pay more attention to the evaluation of ADE *in vitro* and *in vivo* during the development or quality control process. Fc engineering and antibody cocktails are two suitable strategies to deal with the ADE problem. Future studies** s**hould also track SARS-CoV-2 variants, especially antibody-resistant variants. Developing monoclonal antibodies with more diverse targets and formulating cocktail antibodies more flexibly may be proper strategies. In addition, future studies should keep an eye on the development of a special COVID-19 therapeutics antibody database with more comprehensive data fields and better bioinformatics tools for the evaluation of antibody developability. We believe therapeutic antibodies against COVID-19 will play an even more important role in the fight against the catastrophic pandemic in the future.

## Figures and Tables

**Table 1:** Anti-virus monoclonal antibodies for COVID-19 treatment at or after phase 3 trials

Name	Target	Status	Developer
Bamlanivimab	Spike protein	EUA (USA)	Eli Lilly
Sotrovimab	Spike protein	Phase 3	Vir biotechnology/GSK
Regdanvimab	Spike protein	Phase 3	Celltrion
TY027	Spike protein	Phase 3	Tychan

**Table 2:** Anti-host monoclonal antibodies for COVID-19 treatment at or after phase 3 trials

Name	Target	Status	Developer
Levilimab	IL-6R	Approved (Russia)	BIOCAD
Itolizumab	CD6	EUA (India)	Biocon
Tocilizumab	IL-6R	Phase 4	Roche
Ravulizumab-cwvz	C5	Phase 4	Alexion Pharmaceuticals
Sarilumab	IL-6R	Phase 4	Regeneron
Olokizumab	IL-6	Phase 3	R-Pharm JSC/Cromos Pharma
Siltuximab	IL-6	Phase 3	University Hospital, Ghent
Clazakizumab	IL-6	Phase 3	Medical University of Vienna
Mavrilimumab	GM-CSF receptor	Phase 3	Kiniksa Pharmaceuticals
Lenzilumab	GM-CSF	Phase 3	Humanigen
Canakinumab	IL-1β	Phase 3	Novartis
Leronlimab	CCR5	Phase 3	CytoDyn
Emapalumab	IFN gamma	Phase 3	Swedish Orphan Biovitrum
Bevacizumab	VEGF	Phase 3	Roche
IFX-1(BDB-001)	C5a	Phase 3	Staidson/InflaRx
Pamrevlumab	CCN2	Phase 3	FibroGen, Inc.

**Table 3:** Therapeutic cocktail antibodies for COVID-19 treatment

Name	Formulation	Status	Developer	References
REGN-COV2	REGN10933/Casirivimab REGN10987/Imdevimab	EUA (FDA)	Regeneron	73, 108
N.A.	LY-CoV555/bamlanivimab JS016/etesevimab	EUA (FDA)	Eli Lilly	77, 105
AZD7442	AZD8895/Tixagevimab AZD1061/Cilgavimab	Phase 3	AstraZeneca	106
BRII	BRII-196 and BRII-198	Phase 3	Brii Biosciences	40
ADM03820	N.A.	Phase 1	Ology Bioservices	40
N.A.	REGN10989+REGN10934	Preclinical	Regeneron	119
N.A.	REGN10989+REGN10987	Preclinical	Regeneron	119
N.A.	S2E12 + S2M11	Preclinical	UW	120
YH007	Ab1 and Ab5	Preclinical	Biocytogen	40

## References

[1] Zhou P, Yang X Lou, Wang XG, Hu B, Zhang L, Zhang W (2020). A pneumonia outbreak associated with a new coronavirus of probable bat origin. Nature.

[2] Wu F, Zhao S, Yu B, Chen YM, Wang W, Song ZG (2020). A new coronavirus associated with human respiratory disease in China. Nature.

[3] Harrison AG, Lin T, Wang P (2020). Mechanisms of SARS-CoV-2 Transmission and Pathogenesis. Trends Immunol.

[4] Bedford J, Enria D, Giesecke J, Heymann DL, Ihekweazu C, Kobinger G (2020). COVID-19: towards controlling of a pandemic. Lancet.

[5] WHO WHO Coronavirus Disease (COVID-19) Dashboard [Internet]. Available from: https://covid19.who.int.

[6] Hartenian E, Nandakumar D, Lari A, Ly M, Tucker JM, Glaunsinger BA (2020). The molecular virology of coronaviruses. J Biol Chem.

[7] Wang Q, Zhang Y, Wu L, Niu S, Song C, Zhang Z (2020). Structural and Functional Basis of SARS-CoV-2 Entry by Using Human ACE2. Cell.

[8] Lan J, Ge J, Yu J, Shan S, Zhou H, Fan S (2020). Structure of the SARS-CoV-2 spike receptor-binding domain bound to the ACE2 receptor. Nature.

[9] Shang J, Wan Y, Luo C, Ye G, Geng Q, Auerbach A (2020). Cell entry mechanisms of SARS-CoV-2. Proc Natl Acad Sci U S A.

[10] Shang J, Ye G, Shi K, Wan Y, Luo C, Aihara H (2020). Structural basis of receptor recognition by SARS-CoV-2. Nature.

[11] Yan R, Zhang Y, Li Y, Xia L, Guo Y, Zhou Q (2020). Structural basis for the recognition of SARS-CoV-2 by full-length human ACE2. Science.

[12] Michel CJ, Mayer C, Poch O, Thompson JD (2020). Characterization of accessory genes in coronavirus genomes. Virol J.

[13] Kim D, Lee J-Y, Yang J-S, Kim JW, Kim VN, Chang H (2020). The Architecture of SARS-CoV-2 Transcriptome. Cell.

[14] Bojkova D, Klann K, Koch B, Widera M, Krause D, Ciesek S (2020). Proteomics of SARS-CoV-2-infected host cells reveals therapy targets. Nature.

[15] Finkel Y, Mizrahi O, Nachshon A, Weingarten-Gabbay S, Morgenstern D, Yahalom-Ronen Y (2021). The coding capacity of SARS-CoV-2. Nature.

[16] Gordon DE, Jang GM, Bouhaddou M, Xu J, Obernier K, White KM (2020). A SARS-CoV-2 protein interaction map reveals targets for drug repurposing. Nature.

[17] Gordon DE, Hiatt J, Bouhaddou M, Rezelj V V, Ulferts S, Braberg H (2020). Comparative host-coronavirus protein interaction networks reveal pan-viral disease mechanisms. Science.

[18] Li J, Guo M, Tian X, Wang X, Yang X, Wu P (2021). Virus-Host Interactome and Proteomic Survey Reveal Potential Virulence Factors Influencing SARS-CoV-2 Pathogenesis. Med.

[19] Zhou N, Bao J, Ning Y (2021). H2V: a database of human genes and proteins that respond to SARS-CoV-2, SARS-CoV, and MERS-CoV infection. BMC Bioinformatics.

[20] Hoffmann M, Kleine-Weber H, Schroeder S, Krüger N, Herrler T, Erichsen S (2020). SARS-CoV-2 Cell Entry Depends on ACE2 and TMPRSS2 and Is Blocked by a Clinically Proven Protease Inhibitor. Cell.

[21] Johnson BA, Xie X, Bailey AL, Kalveram B, Lokugamage KG, Muruato A (2021). Loss of furin cleavage site attenuates SARS-CoV-2 pathogenesis. Nature.

[22] Wang K, Chen W, Zhang Z, Deng Y, Lian J-Q, Du P (2020). CD147-spike protein is a novel route for SARS-CoV-2 infection to host cells. Signal Transduct Target Ther.

[23] Daly JL, Simonetti B, Klein K, Chen K-E, Williamson MK, Antón-Plágaro C (2020). Neuropilin-1 is a host factor for SARS-CoV-2 infection. Science.

[24] Sigrist CJ, Bridge A, Le Mercier P (2020). A potential role for integrins in host cell entry by SARS-CoV-2. Antiviral Res.

[25] Zhang X, Tan Y, Ling Y, Lu G, Liu F, Yi Z (2020). Viral and host factors related to the clinical outcome of COVID-19. Nature.

[26] Tang Y, Liu J, Zhang D, Xu Z, Ji J, Wen C (2020). Cytokine Storm in COVID-19: The Current Evidence and Treatment Strategies. Front Immunol.

[27] Hu B, Huang S, Yin L (2021). The cytokine storm and COVID-19. J Med Virol.

[28] Zhang L, Lin D, Sun X, Curth U, Drosten C, Sauerhering L (2020). Crystal structure of SARS-CoV-2 main protease provides a basis for design of improved α-ketoamide inhibitors. Science.

[29] Jin Z, Du X, Xu Y, Deng Y, Liu M, Zhao Y (2020). Structure of M pro from COVID-19 virus and discovery of its inhibitors. Nature.

[30] Dai W, Zhang B, Jiang X-M, Su H, Li J, Zhao Y (2020). Structure-based design of antiviral drug candidates targeting the SARS-CoV-2 main protease. Science.

[31] Yin W, Mao C, Luan X, Shen D-D, Shen Q, Su H (2020). Structural basis for inhibition of the RNA-dependent RNA polymerase from SARS-CoV-2 by remdesivir. Science.

[32] Gao Y, Yan L, Huang Y, Liu F, Zhao Y, Cao L (2020). Structure of the RNA-dependent RNA polymerase from COVID-19 virus. Science.

[33] Zhou Y, Wang F, Tang J, Nussinov R, Cheng F (2020). Artificial intelligence in COVID-19 drug repurposing. Lancet Digit Heal.

[34] RECOVERY Collaborative Group, Horby P, Lim WS, Emberson JR, Mafham M, Bell JL (2020). Dexamethasone in Hospitalized Patients with Covid-19 — Preliminary Report. N Engl J Med.

[35] WHO Solidarity Trial Consortium, Pan H, Peto R, Henao-Restrepo A-M, Preziosi M-P, Sathiyamoorthy V (2020). Repurposed Antiviral Drugs for Covid-19 - Interim WHO Solidarity Trial Results. N Engl J Med.

[36] Zhou G, Zhao Q (2020). Perspectives on therapeutic neutralizing antibodies against the Novel Coronavirus SARS-CoV-2. Int J Biol Sci.

[37] Ku Z, Ye X, Salazar GT, Zhang N, An Z (2020). Antibody therapies for the treatment of COVID-19. Antib Ther.

[38] Huang Y, Sun H, Yu H, Li S, Zheng Q, Xia N (2020). Neutralizing antibodies against SARS-CoV-2: current understanding, challenge and perspective. Antib Ther.

[39] Lu L, Zhang H, Zhan M, Jiang J, Yin H, Dauphars DJ (2020). Antibody response and therapy in COVID-19 patients: what can be learned for vaccine development?. Sci China Life Sci.

[40] Yang L, Liu W, Yu X, Wu M, Reichert JM, Ho M (2020). COVID-19 antibody therapeutics tracker: a global online database of antibody therapeutics for the prevention and treatment of COVID-19. Antib Ther.

[41] Duan K, Liu B, Li C, Zhang H, Yu T, Qu J (2020). Effectiveness of convalescent plasma therapy in severe COVID-19 patients. Proc Natl Acad Sci.

[42] Xu X, Han M, Li T, Sun W, Wang D, Fu B (2020). Effective treatment of severe COVID-19 patients with tocilizumab. Proc Natl Acad Sci.

[43] Ye M, Fu D, Ren Y, Wang F, Wang D, Zhang F (2020). Treatment with convalescent plasma for COVID-19 patients in Wuhan, China. J Med Virol.

[44] Rajendran K, Krishnasamy N, Rangarajan J, Rathinam J, Natarajan M, Ramachandran A (2020). Convalescent plasma transfusion for the treatment of COVID-19: Systematic review. J Med Virol.

[45] Salazar E, Perez KK, Ashraf M, Chen J, Castillo B, Christensen PA Treatment of Coronavirus Disease 2019 (COVID-19) Patients with Convalescent Plasma. Am J Pathol. 2020.

[46] Piechotta V, Chai KL, Valk SJ, Doree C, Monsef I, Wood EM (2020). Convalescent plasma or hyperimmune immunoglobulin for people with COVID-19: a living systematic review. Cochrane database Syst Rev.

[47] Li L, Zhang W, Hu Y, Tong X, Zheng S, Yang J (2020). Effect of Convalescent Plasma Therapy on Time to Clinical Improvement in Patients With Severe and Life-threatening COVID-19. JAMA.

[48] Agarwal A, Mukherjee A, Kumar G, Chatterjee P, Bhatnagar T, Malhotra P (2020). Convalescent plasma in the management of moderate covid-19 in adults in India: open label phase II multicentre randomised controlled trial (PLACID Trial). BMJ.

[49] Simonovich VA, Burgos Pratx LD, Scibona P, Beruto M V, Vallone MG, Vázquez C (2020). A Randomized Trial of Convalescent Plasma in Covid-19 Severe Pneumonia. N Engl J Med.

[50] Liu STH, Lin H-M, Baine I, Wajnberg A, Gumprecht JP, Rahman F (2020). Convalescent plasma treatment of severe COVID-19: a propensity score-matched control study. Nat Med.

[51] Salazar E, Christensen PA, Graviss EA, Nguyen DT, Castillo B, Chen J (2020). Treatment of Coronavirus Disease 2019 Patients with Convalescent Plasma Reveals a Signal of Significantly Decreased Mortality. Am J Pathol.

[52] Salazar E, Christensen PA, Graviss EA, Nguyen DT, Castillo B, Chen J (2021). Significantly Decreased Mortality in a Large Cohort of Coronavirus Disease 2019 (COVID-19) Patients Transfused Early with Convalescent Plasma Containing High-Titer Anti-Severe Acute Respiratory Syndrome Coronavirus 2 (SARS-CoV-2) Spike Protein IgG. Am J Pathol.

[53] Libster R, Pérez Marc G, Wappner D, Coviello S, Bianchi A, Braem V (2021). Early High-Titer Plasma Therapy to Prevent Severe Covid-19 in Older Adults. N Engl J Med.

[54] Joyner MJ, Carter RE, Senefeld JW, Klassen SA, Mills JR, Johnson PW (2021). Convalescent Plasma Antibody Levels and the Risk of Death from Covid-19. N Engl J Med.

[55] Katz LM (2021). (A Little) Clarity on Convalescent Plasma for Covid-19. N Engl J Med.

[56] Jawhara S (2020). Could Intravenous Immunoglobulin Collected from Recovered Coronavirus Patients Protect against COVID-19 and Strengthen the Immune System of New Patients?. Int J Mol Sci.

[57] Cao W, Liu X, Bai T, Fan H, Hong K, Song H (2020). High-Dose Intravenous Immunoglobulin as a Therapeutic Option for Deteriorating Patients With Coronavirus Disease 2019. Open Forum Infect Dis.

[58] Shao Z, Feng Y, Zhong L, Xie Q, Lei M, Liu Z (2020). Clinical efficacy of intravenous immunoglobulin therapy in critical ill patients with COVID-19: a multicenter retrospective cohort study. Clin Transl Immunol.

[59] Zhang J, Yang Y, Yang N, Ma Y, Zhou Q, Li W (2020). Effectiveness of intravenous immunoglobulin for children with severe COVID-19: a rapid review. Ann Transl Med.

[60] Todd PA, Brogden RN (1989). Muromonab CD3. Drugs.

[61] Mac S, Sumner A, Duchesne-Belanger S, Stirling R, Tunis M, Sander B (2019). Cost-effectiveness of Palivizumab for Respiratory Syncytial Virus: A Systematic Review. Pediatrics.

[62] Desoubeaux G, Pelegrin M (2019). Monoclonal antibodies in infectious diseases: new partners in the therapeutic arsenal. Med Sci.

[63] Marston HD, Paules CI, Fauci AS (2018). Monoclonal Antibodies for Emerging Infectious Diseases — Borrowing from History. N Engl J Med.

[64] Wan J, Xing S, Ding L, Wang Y, Gu C, Wu Y (2020). Human-IgG-Neutralizing Monoclonal Antibodies Block the SARS-CoV-2 Infection. Cell Rep.

[65] Pinto D, Park Y-J, Beltramello M, Walls AC, Tortorici MA, Bianchi S (2020). Cross-neutralization of SARS-CoV-2 by a human monoclonal SARS-CoV antibody. Nature.

[66] Wu NC, Yuan M, Liu H, Lee C-CD, Zhu X, Bangaru S (2020). An Alternative Binding Mode of IGHV3-53 Antibodies to the SARS-CoV-2 Receptor Binding Domain. Cell Rep.

[67] Huo J, Zhao Y, Ren J, Zhou D, Duyvesteyn HME, Ginn HM (2020). Neutralization of SARS-CoV-2 by Destruction of the Prefusion Spike. Cell Host Microbe.

[68] Zhou D, Duyvesteyn HME, Chen C-P, Huang C-G, Chen T-H, Shih S-R (2020). Structural basis for the neutralization of SARS-CoV-2 by an antibody from a convalescent patient. Nat Struct Mol Biol.

[69] Cao Y, Su B, Guo X, Sun W, Deng Y, Bao L (2020). Potent Neutralizing Antibodies against SARS-CoV-2 Identified by High-Throughput Single-Cell Sequencing of Convalescent Patients' B Cells. Cell.

[70] Yuan M, Liu H, Wu NC, Lee C-CD, Zhu X, Zhao F (2020). Structural basis of a shared antibody response to SARS-CoV-2. Science.

[71] Rogers TF, Zhao F, Huang D, Beutler N, Burns A, He W (2020). Isolation of potent SARS-CoV-2 neutralizing antibodies and protection from disease in a small animal model. Science.

[72] Yuan M, Wu NC, Zhu X, Lee C-CD, So RTY, Lv H (2020). A highly conserved cryptic epitope in the receptor binding domains of SARS-CoV-2 and SARS-CoV. Science.

[73] Hansen J, Baum A, Pascal KE, Russo V, Giordano S, Wloga E (2020). Studies in humanized mice and convalescent humans yield a SARS-CoV-2 antibody cocktail. Science.

[74] Hurlburt NK, Seydoux E, Wan Y-H, Edara VV, Stuart AB, Feng J (2020). Structural basis for potent neutralization of SARS-CoV-2 and role of antibody affinity maturation. Nat Commun.

[75] Lv Z, Deng Y-Q, Ye Q, Cao L, Sun C-Y, Fan C (2020). Structural basis for neutralization of SARS-CoV-2 and SARS-CoV by a potent therapeutic antibody. Science.

[76] Wang C, Li W, Drabek D, Okba NMA, van Haperen R, Osterhaus ADME (2020). A human monoclonal antibody blocking SARS-CoV-2 infection. Nat Commun.

[77] Shi R, Shan C, Duan X, Chen Z, Liu P, Song J (2020). A human neutralizing antibody targets the receptor-binding site of SARS-CoV-2. Nature.

[78] Barnes CO, Jette CA, Abernathy ME, Dam K-MA, Esswein SR, Gristick HB (2020). SARS-CoV-2 neutralizing antibody structures inform therapeutic strategies. Nature.

[79] Barnes CO, West AP, Huey-Tubman KE, Hoffmann MAG, Sharaf NG, Hoffman PR (2020). Structures of Human Antibodies Bound to SARS-CoV-2 Spike Reveal Common Epitopes and Recurrent Features of Antibodies. Cell.

[80] Ejemel M, Li Q, Hou S, Schiller ZA, Tree JA, Wallace A (2020). A cross-reactive human IgA monoclonal antibody blocks SARS-CoV-2 spike-ACE2 interaction. Nat Commun.

[81] Hassan AO, Case JB, Winkler ES, Thackray LB, Kafai NM, Bailey AL (2020). A SARS-CoV-2 Infection Model in Mice Demonstrates Protection by Neutralizing Antibodies. Cell.

[82] Ju B, Zhang Q, Ge J, Wang R, Sun J, Ge X (2020). Human neutralizing antibodies elicited by SARS-CoV-2 infection. Nature.

[83] Seydoux E, Homad LJ, MacCamy AJ, Parks KR, Hurlburt NK, Jennewein MF (2020). Analysis of a SARS-CoV-2-Infected Individual Reveals Development of Potent Neutralizing Antibodies with Limited Somatic Mutation. Immunity.

[84] Chi X, Yan R, Zhang J, Zhang G, Zhang Y, Hao M (2020). A neutralizing human antibody binds to the N-terminal domain of the Spike protein of SARS-CoV-2. Science.

[85] Wrapp D, De Vlieger D, Corbett KS, Torres GM, Wang N, Van Breedam W (2020). Structural Basis for Potent Neutralization of Betacoronaviruses by Single-Domain Camelid Antibodies. Cell.

[86] Hwang WC, Lin Y, Santelli E, Sui J, Jaroszewski L, Stec B (2006). Structural Basis of Neutralization by a Human Anti-severe Acute Respiratory Syndrome Spike Protein Antibody, 80R. J Biol Chem.

[87] Hanke L, Vidakovics Perez L, Sheward DJ, Das H, Schulte T, Moliner-Morro A (2020). An alpaca nanobody neutralizes SARS-CoV-2 by blocking receptor interaction. Nat Commun.

[88] Huo J, Le Bas A, Ruza RR, Duyvesteyn HME, Mikolajek H, Malinauskas T (2020). Neutralizing nanobodies bind SARS-CoV-2 spike RBD and block interaction with ACE2. Nat Struct Mol Biol.

[89] Sun Y, Ho M (2020). Emerging antibody-based therapeutics against SARS-CoV-2 during the global pandemic. Antib Ther.

[90] Chen P, Nirula A, Heller B, Gottlieb RL, Boscia J, Morris J (2021). SARS-CoV-2 Neutralizing Antibody LY-CoV555 in Outpatients with Covid-19. N Engl J Med.

[91] Mahase E (2020). Covid-19: FDA authorises neutralising antibody bamlanivimab for non-admitted patients. BMJ.

[92] Chan KK, Dorosky D, Sharma P, Abbasi SA, Dye JM, Kranz DM (2020). Engineering human ACE2 to optimize binding to the spike protein of SARS coronavirus 2. Science.

[93] Kruse RL (2020). Therapeutic strategies in an outbreak scenario to treat the novel coronavirus originating in Wuhan, China. F1000Research.

[94] Miao X, Luo Y, Huang X, Lee SMY, Yuan Z, Tang Y (2020). A novel biparatopic hybrid antibody-ACE2 fusion that blocks SARS-CoV-2 infection: implications for therapy. MAbs.

[95] Li Y, Wang H, Tang X, Fang S, Ma D, Du C (2020). SARS-CoV-2 and Three Related Coronaviruses Utilize Multiple ACE2 Orthologs and Are Potently Blocked by an Improved ACE2-Ig. J Virol.

[96] Zhang L, Zheng B, Gao X, Zhang L, Pan H, Qiao Y (2020). Development of Patient-Derived Human Monoclonal Antibodies Against Nucleocapsid Protein of Severe Acute Respiratory Syndrome Coronavirus 2 for Coronavirus Disease 2019 Diagnosis. Front Immunol.

[97] Cantuti-Castelvetri L, Ojha R, Pedro LD, Djannatian M, Franz J, Kuivanen S (2020). Neuropilin-1 facilitates SARS-CoV-2 cell entry and infectivity. Science.

[98] Bian H, Zheng ZH, Wei D, Zhang Z, Kang WZ, Hao CQ (2020). Meplazumab treats COVID-19 pneumonia: An open-labelled, concurrent controlled add-on clinical trial. medRxiv.

[99] Xia P, Dubrovska A (2020). Tumor markers as an entry for SARS-CoV-2 infection?. FEBS J.

[100] Guaraldi G, Meschiari M, Cozzi-Lepri A, Milic J, Tonelli R, Menozzi M (2020). Tocilizumab in patients with severe COVID-19: a retrospective cohort study. Lancet Rheumatol.

[101] Stone JH, Frigault MJ, Serling-Boyd NJ, Fernandes AD, Harvey L, Foulkes AS (2020). Efficacy of Tocilizumab in Patients Hospitalized with Covid-19. N Engl J Med.

[102] Salama C, Han J, Yau L, Reiss WG, Kramer B, Neidhart JD (2021). Tocilizumab in Patients Hospitalized with Covid-19 Pneumonia. N Engl J Med.

[103] Ho M (2020). Perspectives on the development of neutralizing antibodies against SARS-CoV-2. Antib Ther.

[104] Qiu X, Wong G, Audet J, Bello A, Fernando L, Alimonti JB (2014). Reversion of advanced Ebola virus disease in nonhuman primates with ZMapp. Nature.

[105] Gottlieb RL, Nirula A, Chen P, Boscia J, Heller B, Morris J (2021). Effect of Bamlanivimab as Monotherapy or in Combination With Etesevimab on Viral Load in Patients With Mild to Moderate COVID-19: A Randomized Clinical Trial. JAMA.

[106] Zost SJ, Gilchuk P, Case JB, Binshtein E, Chen RE, Nkolola JP (2020). Potently neutralizing and protective human antibodies against SARS-CoV-2. Nature.

[107] Baum A, Ajithdoss D, Copin R, Zhou A, Lanza K, Negron N (2020). REGN-COV2 antibodies prevent and treat SARS-CoV-2 infection in rhesus macaques and hamsters. Science.

[108] Weinreich DM, Sivapalasingam S, Norton T, Ali S, Gao H, Bhore R (2021). REGN-COV2, a Neutralizing Antibody Cocktail, in Outpatients with Covid-19. N Engl J Med.

[109] Rick Mullin (2020). AstraZeneca picks Lonza for COVID drug. C&EN Glob Enterp.

[110] Wen J, Cheng Y, Ling R, Dai Y, Huang B, Huang W (2020). Antibody-dependent enhancement of coronavirus. Int J Infect Dis.

[111] Wan Y, Shang J, Sun S, Tai W, Chen J, Geng Q (2020). Molecular Mechanism for Antibody-Dependent Enhancement of Coronavirus Entry. J Virol.

[112] Hikmet F, Méar L, Edvinsson Å, Micke P, Uhlén M, Lindskog C (2020). The protein expression profile of ACE2 in human tissues. Mol Syst Biol.

[113] Ren X, Wen W, Fan X, Hou W, Su B, Cai P (2021). COVID-19 immune features revealed by a large-scale single cell transcriptome atlas. Cell. 2021; Available from: https://doi.org/10.1016/j.cell.

[114] Arvin AM, Fink K, Schmid MA, Cathcart A, Spreafico R, Havenar-Daughton C (2020). A perspective on potential antibody-dependent enhancement of SARS-CoV-2. Nature.

[115] Lee WS, Wheatley AK, Kent SJ, DeKosky BJ (2020). Antibody-dependent enhancement and SARS-CoV-2 vaccines and therapies. Nat Microbiol.

[116] Plante JA, Liu Y, Liu J, Xia H, Johnson BA, Lokugamage KG (2020). Spike mutation D614G alters SARS-CoV-2 fitness. Nature.

[117] Weisblum Y, Schmidt F, Zhang F, DaSilva J, Poston D, Lorenzi JCC (2020). Escape from neutralizing antibodies by SARS-CoV-2 spike protein variants. Elife.

[118] Lauring AS, Hodcroft EB (2021). Genetic Variants of SARS-CoV-2-What Do They Mean?. JAMA.

[119] Baum A, Fulton BO, Wloga E, Copin R, Pascal KE, Russo V (2020). Antibody cocktail to SARS-CoV-2 spike protein prevents rapid mutational escape seen with individual antibodies. Science.

[120] Tortorici MA, Beltramello M, Lempp FA, Pinto D, Dang H V, Rosen LE (2020). Ultrapotent human antibodies protect against SARS-CoV-2 challenge via multiple mechanisms. Science (80- ).

[121] Brainard J (2020). Scientists are drowning in COVID-19 papers. Can new tools keep them afloat? Science.

[122] Roos DS (2001). Bioinformatics-Trying to Swim in a Sea of Data. Science.

[123] Dzisoo AM, He B, Karikari R, Agoalikum E, Huang J (2019). CISI: A Tool for Predicting Cross-interaction or Self-interaction of Monoclonal Antibodies Using Sequences. Interdiscip Sci Comput Life Sci.

[124] Dzisoo AM, Kang J, Yao P, Klugah-Brown B, Mengesha BA, Huang J (2020). SSH: A Tool for Predicting Hydrophobic Interaction of Monoclonal Antibodies Using Sequences. Biomed Res Int.

